# The Infectious Pancreatic Necrosis Virus (IPNV) and its Virulence Determinants: What is Known and What Should be Known

**DOI:** 10.3390/pathogens9020094

**Published:** 2020-02-04

**Authors:** Carlos P. Dopazo

**Affiliations:** Instituto de Acuicultura, Departamento de Microbiología y Parasitología, Universidade de Santiago de Compostela, 15782 Santiago de Compostela, Spain; carlos.pereira@usc.es

**Keywords:** IPNV, virulence

## Abstract

Infectious pancreatic necrosis (IPN) is a disease of great concern in aquaculture, mainly among salmonid farmers, since losses in salmonid fish—mostly very young rainbow trout (*Salmo gairdnery*) fry and Atlantic salmon (*Salmo salar*) post-smolt—frequently reach 80–90% of stocks. The virus causing the typical signs of the IPN disease in salmonids, named infectious pancreatic necrosis virus (IPNV), has also been isolated from other fish species either suffering related diseases (then named IPNV-like virus) or asymptomatic; the general term aquabirnavirus is used to encompass all these viruses. Aquabirnaviruses are non-enveloped, icosahedral bisegmented dsRNA viruses, whose genome codifies five viral proteins, three of which are structural, and one of them is an RNA-dependent RNA polymerase. Due to the great importance of the disease, there have been great efforts to find a way to predict the level of virulence of IPNV isolates. The viral genome and proteins have been the main focus of research. However, to date such a reliable magic marker has not been discovered. This review describes the processes followed for decades in the attempts to discover the viral determinants of virulence, and to help the reader understand how viral components can be involved in virulence modulation in vitro and in vivo. There is also a brief description of the disease, of host defenses, and of the molecular structure and function of the virus and its viral components.

## 1. Introduction

Infectious pancreatic necrosis (IPN) is a well-known disease originally named acute catarrhal enteritis by M’Gonigle in 1951 [1], but soon after Wood et al. [2] changed its name to IPN, based on a histopathological study of brook trout (*Salvelinus fontinalis*) suffering an infectious disease resembling a catarrhal enteritis. The virus causing the disease, the IPN virus (IPNV), was the first fish virus isolated in vitro [3] and belongs to the family *Birnaviridae* and the genus *Aquabirnavirus.* Although originally isolated from brown trout and considered a disease of great impact on cultured salmonids worldwide [4], it has also been isolated from non-salmonid diseased fishes, as well as from a wide range of fish species from natural environments [5,6,7]. In fact, the term IPNV is strictly used for those strains affecting salmonid fish which develop specific symptoms (see below). In those cases where the virus affects non-salmonids, with different symptoms, the term IPN is substituted by IPNV-like, and in general terms the name aquabirnaviruses is employed.

The disease mainly affects very young fry; salmonids are mostly susceptible immediately after yolk sac absorption and the first feeding. The clinical symptoms of the disease, described by many researchers since the first in-depth review by Wolf [8], include general signs that are quite common to other viruses, but also a very specific one: abnormal erratic corkscrew swimming, which is the most characteristic in salmonid fingerlings affected by the virus. However, anorexia is also a frequent sign of the disease in salmon post-smolt and in other non-salmonid species.

Although both the disease and the virus are considered to be distributed worldwide, the high numbers of reports published on this virus are not equally distributed. In fact, the web site of the Instituto de Acuicultura from the University of Santiago de Compostela, Spain, (www.usc.es/gl/institutos/acuicultura/difusion/aportacions-cientificas.html) includes a set of IPNV epidemiology maps (and a spreadsheet with a list of references) highlighting the great effort that specialists have made to understand how the virus is distributed worldwide, even though it is still incomplete. In fact, although most reported episodes and isolations were initially in North America and Europe (from a large number of countries), many cases from Asian countries (including Japan, Korea, Taiwan, Iran, Turkey, and China) have been reported since the 1980s. Other areas, such as South America (Chile), Africa (Kenya), and Australia are much less represented.

Due to the significant economic impact of the disease on the aquaculture industry, great effort has been made to control IPN by means of different approaches: early diagnosis and risk assessment; control of trade movements; improving epidemiological knowledge (screening of natural populations; control of carriers; viral types world distribution); and designing vaccines. In any case, an in-depth knowledge of the parameters affecting and modulating the level of virulence of the IPNV virus (and hence of its level of risk) is needed, and many researchers have focused their studies on that subject. We have prepared a review of all the research that has been carried out since the first isolation of the virus, to understand and predict its virulence. But first, a brief description of the disease and the virus has been included to better understand how virulence can be modulated by the different determinants described in the literature.

## 2. The Virus

### 2.1. Its Structure and General Characteristics

The IPNV is an unenveloped icosahedral virus with an average size of around 65 nm, as stated by the International Committee for Taxonomy of Viruses (ICTV) for the general characteristics of the family *Birnaviridae* [9], or 60 nm as reviewed by Munro and Midtlyng [4]. However, a diameter ranging from 57 to 74 nm was reported in an early review by Dobos and Roberts [10], which is more in accordance with the results of a recent study by Lago et al. [11], where a range of sizes between 55 and even 90 nm were visualized in different fractions of a purified IPNV West Buxton type virus (and other type strains), the most frequently observed size being around 70 nm. The virion, of a molecular weight of 55 × 10^6^ Da, shows an approximate protein/RNA content rate of 91/9, a buoyant density in CsCl of 1.33g/ml, and a sedimentation coefficient of 435 S. But one of the main features of this virus is its high stability to physicochemical conditions: pH (stable at pH values as low as 3), salinity (from 0‰ to 40‰), and temperature (resistant to up to 60 °C for 30 min, and able to replicate from 4 to 27.5 °C) as reviewed by Wolf [8] and more recently by Munro and Midtlyng [4].

Regarding the classification of IPNV strains or—in general terms of aquatic birnaviruses—two approaches are applied (see revisions [4,5]). The first classification, based on serological typing, was definitively outlined by Hill and Way [12]. They classified the aquabirnaviruses into two serogroups (A and B) and nine serotypes within serogroup A (Table 1). For the second approach, in spite of the high number of reports providing a diverse classification [4], there is a consensus to consider as definitive the typing into six genogroups proposed by Blake et al. [13] and later extended by Nishizawa et al. [14] with a seventh (Table 1). There is a correspondence between serotype, genotype, and type strain: the American strains WB (West Buxton) and Ja (Jasper), from USA and Canada, respectively, would constitute genotype 1, corresponding to serotypes A1 and A9, respectively; the Danish type strains Sp (Spajarup) and Ab (Abildt) constitute serotypes A2 and A3, and genotypes 5 and 2, respectively; Hetch (He), originally from Germany, is serotype A4 and genotype 6; genotype 3 is constituted by isolates clustering with type strains Te (Tellina, from UK; serotype A5) and C1 (Canada 1; serotype A6), and type strains C3 and C5 (from Canada, corresponding to serotypes A7 and A8) constitute genotype 4; finally, the seventh genotype corresponds to Japanese marine birnavirus (MaBV).

### 2.2. The Viral Genome

**Figure 1:**
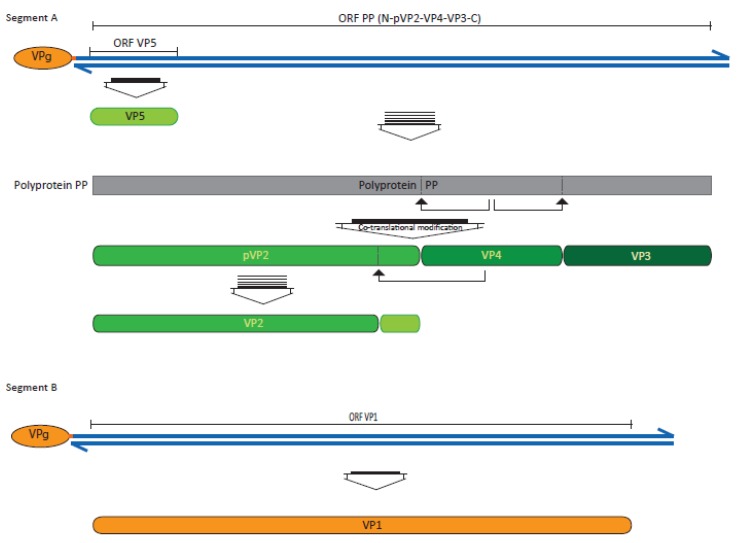
Genome organization and expression.

The genome is constituted by two segments of dsRNA named A and B. Although the virion was traditionally thought to contain a single set of both segments, it is now known to be polyploid, able to package 1, 1.5, and even 2 genome equivalents [11]. Segment A, with a size between 2962 and 3097 bp [4], contains two open reading frames (ORF; Figure 1). The ORF PP, the largest one, encodes a polyprotein of around 106 kDa, which comprises most of the viral proteins: NH_2_-pVP2-VP4-VP3-COOH. Due to the internal proteolytic activity of the VP4 region, this polyprotein suffers a co-translational modification consisting in a protease cleavage, between an alanine and a serine, in two positions: between amino acids 734 and 735, to release the minor capsid protein VP3, and between amino acids 508 and 509, to produce the non-structural viral protein VP4 and pVP2; this is an immature precursor of the major structural protein VP2 which, during morphogenesis, suffers proteolytic cleavage creating the mature structural form and three additional small peptides which remain associated to the virion [15]. The second ORF of this segment, ORF VP5, codifying the non-structural VP5 protein, is in fact the first one since it overlaps ORF PP and its start codon precedes the ORF PP start codon by a few nucleotides. Finally, segment B, of around 2400 bp, contains a single ORF encoding the minor structural protein VP1.

The 5′- and 3′-ends of the viral genome have untranslated terminal repeats (5′- and 3′-UTR) with an important implication in several steps of the viral replication, as well as in virulence. To this regard, as first reported by Dobos [16] and Magyar et al. [17], the 5′-ends of both segments are the sites for the covalent cell attachment of the structural VP1 protein, which, in this attached form, is named VPg to differentiate it from the free VP1 form. Mutations in this 5′-UTR were known to affect IPNV infectivity [18], and Boot et al. [19] suggested that, since birnaviruses lack a 5′-cap and—because of the short length of the 5′-UTR—also lack an internal ribosome entry site (IRES) to use the cell-encoded initiation factors, the VPg linked at the 5′-end would be involved in initiation of translation. However, it has also been reported that 5′-UTR lengths shorter than the normal 300 nt are not necessarily an impediment to constitute an IRES structure, and Rivas-Aravena et al. [20] have demonstrated that the 5′-UTR forms a functional structure, efficiently acting as an IRES, which commands translation.

At the other end of the strands, the 3´-UTR is known to be involved in second strand RNA synthesis. The infectious bursal disease virus (IBDV; a member of the related genus *Avibirnavirus* frequently used as reference for IPNV structure and replication) is known to have a couple of cytosines at this end allowing the VPg to act as a protein-primer thanks to its linked guanines; but to function, the 3′-UTR must maintain a specific stem-loop structure [20]. In the case of the mRNA, since its 3′-end lacks a poly(A) tail to simulate the cellular mRNA and thus to defend against exonuclear activity, and enhance its translation, its function is probably substituted by the 3′-UTR stem loop structure [19].

### 2.3. The Viral Proteins and Their Function

The viral genome encodes five viral proteins named from VP1 to VP5. Three of them (VP1, VP2, and VP3) are structural, and to date two (VP4 and VP5) are believed to be non-structural proteins. An ORF encoding a putative sixth 25 kDa viral protein has been found by Shivappa et al. [21] overlapping part of the ORF PP, between the pVP2 and VP4 sequences. They found that sequence exclusively in the Sp strain, and later a similar sequence was also found in a few Chilean strains. Due to the presence of positively-charged domains, it was speculated to interact with the inner plasmatic membrane leaflet [22]. 

VP1, the viral protein encoded by the genome segment B (with an approximate size of 94 kDa), is the viral RNA-dependent RNA-polymerase (RdRp) needed for genome replication and transcription [23]. As previously described, this structural protein is present in two forms: VP1, free inside the capsid, and VPg, linked to the 5′-ends of both genomes [4], probably only to the plus strands [17].

The same VP1 polypeptide may act as an RdRp and a protein-primer for the initiation of RNA synthesis. For that purpose, VP1 suffers a self-guanylylation, without the need of a template RNA to produce VP1pGpG [24,25]. The cytosines at the 3′-end termini of the genome will allow that molecule to act as the protein-primer for the synthesis of a second RNA strand. Consequently, VP1 remains linked to the 5′-end of the new synthetized strand, thus becoming VPg.

**Figure 2:**
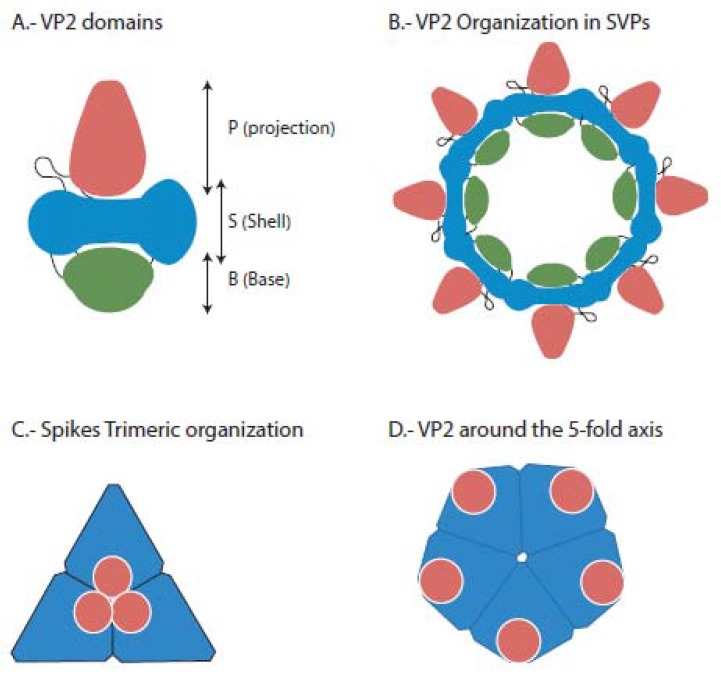
General structure and organization of the VP2 protein in infectious pancreatic necrosis virus (IPNV). (**A**) VP2 is structurally constituted by three domains: a central one (blue), which in subviral particles (SVPs) constitutes the shell (see panel (**B**)) and is named before that (S); the base (B; green), which is located in the inner side of the particle, and the spike or projection (P, red), on the other side. (**B**) Structural organization of VP2 in SVPs. (**C**) Trimeric organization of the spikes. (**D**) Organization of the VP2 units around the 5-fold axis. (Adapted from Caulibaly et al. [28]).

VP2, a structural protein of about 54kDa [5], is, in number of units, the main component of the capsid. Its structure and organization have been recently well defined by Coulibaly et al. [26,27] (Figure 2). It is constituted of three domains (Figure 2A): a central one—called S—which, in subviral particles (SVPs) constitutes the shell (Figure 2B); the base (B), which is located in the inner side of the particle, and the spike or projection (P) to the outside of the capside. The spikes—organized in VP2 trimers around a 3-fold axis (Figure 2C)—contain the main viral antigenic sites, the cell specificity epitope, and some of the virulence determinants, as described in more detail below.

**Figure 3:**
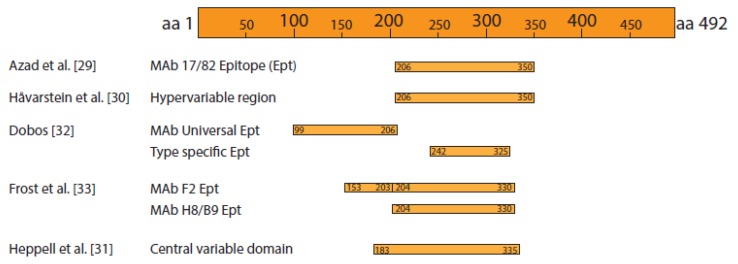
VP2 epitopes and variable region map.

Before that study, VP2 was already known to carry the cell attachment sites and to be responsible for most of the antigenicity of the virus. As early as in the late 1980s, Caswel-Reno et al. [28] demonstrated the presence of neutralization epitopes in VP2, and Azad et al. [29] located the same type of epitopes in an internal region of VP2—between amino acids 206 and 350—which was recognized by the virus-neutralizing monoclonal antibody (MAb) 17/82 (Figure 3). That section of VP2 was soon recognized by Håvarstein et al. [30] to be a hypervariable region in the VP2 sequence, almost coinciding with the widely known “central variable domain” described by Heppell et al. [31]. Other studies using MAbs confirmed VP2 to be the main protein responsible for IPNV antigenicity, and in one such study Dobos [32] found a serotype-specific epitope, also located in that central region of the molecule.

Even prior to discovering its antigenicity [33], VP2 was known to be the viral protein in charge of cell-attachment and was therefore considered responsible for cell and host specificity [34]. The attachment site is located in the P domain of VP2 (Figure 2A) at the top of the spike [27]; however, certain amino acids located at the groove of the spike, close to domain S, are also important for binding to the cell, at least in IBDV [35]. Therefore, Coulibaly et al. [27] suggested that those two units could correspond to two different receptors, the first, at the top of the spike, would be for cell recognition and attachment, and the one in the spike’s groove would oversee cell internalization.

The viral proteins involved in cell attachment are frequently glycosylated, since that type of maturation confers special properties to the viral protein, which are important in the process of adsorption to cells and, therefore, in cell tropism and virulence. There are two types of glycosylation; the most common, the N-linked glycosylation, corresponds to a post-translational modification taking place inside the endoplasmic reticulum (ER); the second one, the O-glycosylation, reported to occur freely in the cytoplasm, has been demonstrated to be important for IPNV integrity and infectivity [30,36,37,38]. 

VP3 is a relatively small molecule with several functions. As reviewed in a recent study where the authors reported that IBDV-VP3 upregulates the RNA synthesis activity by the viral RdRp [39], VP3 is a multifunctional protein which also acts as a scaffolding protein during morphogenesis and provides protection against the anti-viral cell response; additionally, it has been reported to induce apoptosis in fish [40]. Vp3, first thought to be a trimeric [41] and later dimeric [42] protein, is the second component of the capsid in terms of copy numbers, behind VP2, the major one. VP3 is known to be an internal viral protein in IPNV since Dobos [32] discovered that not only the genome but also this protein were absent in empty capsids. Nevertheless, part of the protein must be exposed to the outer side of the capsid since some reaction with neutralizing MAbs has been demonstrated [43]. VP3 interacts with VP1, and also captures the viral genome, constituting a ribonucleoprotein (RNP) structure which protects the viral RNA against the host defenses [44,45]. For this VP3–dsRNA binding, the N-terminal of the protein is crucial, although both the C- and N-end domains are important for the binding [44]. Although Bahar et al. [46] reported that this association had no effect on VP1 activity, soon after Ferrero et al. [39] demonstrated that VP3 did actually have an upregulating effect on the VP1-mediated RNA replication. Additionally, the VP1–VP3 complex promotes the assembly of pVP2 units, constructing a precursor capsid which, after the maturation of pVP2 by proteolysis to VP2 (and three small peptides), will constitute the mature capsid [44,45].

VP4 is the viral protein whose autoproteolytic activity cleaves the polyprotein PP during its own translation. This is VP4’s most known function, but this protein is also known to trans-activate the synthesis of VP1 [47], although the mechanism is still unknown; and, more recently [48] it has been discovered that VP4 also strongly inhibits interferon induction, and such antagonistic activity is not linked to its proteolytic activity.

**Figure 4:**
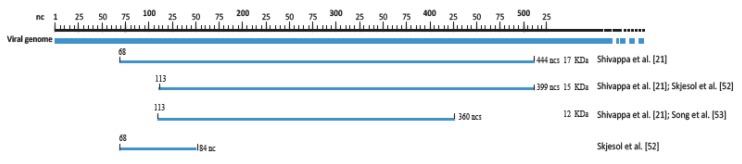
VP5 protein open reading frame (ORF).

VP5 is a non-structural protein codified by the small ORF at the 5′-end of segment A. There is a certain discrepancy about the size of this protein, undoubtedly due to the high variability observed. The size of this protein in IPNV was originally reported to be of 17 kDa [49], similar to that of IBDV [50], although a size of 15 kDa was also reported, since two start codons [51] can appear in the 5′-end sequence of that segment (Figure 4), one in nucleotide (nt) position 68, and a second in nt 112, depending on the strain. However, in 2001 Webber et al. [18] demonstrated that, in IPNV, VP5 used the second start codon position, encoding a 15 kDa (399 nt/133 aa) protein [21,52], although a truncated 12 kDa VP5 has been frequently detected in some strains [21,53]. In addition, a shorter truncated form of only 28 aa, due to the presence of a premature stop-codon, has also been reported [52].

The function of this protein has not been definitively defined yet. Although it is considered non-essential for virus replication both in vitro [18] and in vivo [54], it has been revealed to regulate protein expression in the early steps of viral replication, and even to enhance cell viability by preventing membrane rupture and DNA cleavage, as reported by Hong et al. [55], which in fact enhances progeny production. Such antiapoptotic activity is widely accepted for IBDV–VP5 [56], but there is much controversy surrounding the antiapoptotic function of this protein in IPNV. To this regard, in spite of the homology of IPNV–VP5 with the Bcl-2 antiapoptotic proteins, previously reported [55] and demonstrated by Ortega et al. [57], these authors concluded that VP5 had no influence on apoptosis. This supported previous results by Santi et al. [54]; however, in their paper they recognized that the mutations observed in putatively important locations in the domain of the strains under study could be an explanation for the lack of activity. Therefore, IPNV–VP5 is still accepted as an antiapoptotic protein [58,59], which also has a strong inhibition of the interferon (IFN) signaling [48,60]. Finally, in IBDV, in addition to this antiapoptotic activity at early stages of infection, VP5 has been demonstrated to activate cell apoptosis in late stages, possibly constituting a mechanism for progeny release [61], something that could be similar in IPNV.

### 2.4. Replication Cycle

Understanding the structure of the virus, as we have done above, as well as the function of its components is a prerequisite to recognizing the strategies it uses to modulate virulence.

Therefore, we will now look into the way the virus infects and replicates in a susceptible cell (see reviews [4,62]). A complete replication cycle takes between 16 and 20 h. In the first step, adsorption, VP2 acts as the attachment viral receptor to specifically recognize the surface cell receptor. In CHSE-214 cells, up to 6000 of those receptors have been calculated to be available for IPNV fixation [63], although just a quarter of them would provide a specific attachment. VP2 has two domains involved in adsorption [27], one at the top of the spike, used for specific fixation to the cell receptor, and a second at the groove, close to the bottom of the spike, involved in the internalization step. Adsorption takes around 20 min, internalization is produced via receptor-mediated endocytosis, and in just 2 h p.i. new synthesized RNA (a transcription intermediate) is detected. Since dsRNA is susceptible to being identified as exogenous by the cell defenses, it was thought to be protected like in the case of reovirus infection, which carries out transcription and replication inside a viral core. To this regard, RdRp activity has been associated to IPNV virions without proteolytic treatment [62]. However, an RNP made up by a VP1–VP3–RI (RNA intermediate) complex is also known to be involved in RNA synthesis [44,45]. In fact, both types of RNPs have been demonstrated to play a role in RNA synthesis [64], and to protect the new synthesized viral RNA against cell defenses. The progeny genome synthesis starts between 4 and 6 h p.i. and protein synthesis is also observed during that time; the maximum genome synthesis is reached 8–10 h p.i and declines at 14–16 h p.i. [62]. The VP3 protein in the VP1–VP3–dsRNA RNP complex acts as a scaffolding protein [44,45], capturing pVP2 to construct an immature non-infectious 68 nm particle. This particle, which appears after 8 h p.i., at the beginning of the morphogenesis phase, suffers a maturation process to be transformed (12 h p.i.) into the 60 nm infective virion [65]; this maturation consists of the proteolytic cleavage of pVP2 into VP2 by the activity of VP4, although the participation of cell proteases cannot be dismissed [32]. Finally, if the internalization of the viral progeny is as reported for IBDV [66,67], VP5 would accumulate in the cell membrane, triggering its lysis and the viral release.

### 2.5. IPNV and Persistence

A widely known fact is that the reservoirs of an episode become lifelong asymptomatic carriers, in which the virus can be detected—in certain tissues—mainly after stress episodes. Initially, it was accepted that the virus used a high proportion of defective interfering (DI) particles to create a balance between infective viral particles and host defenses. DI particles have the capacity to attach and penetrate into a susceptible cell, but not to replicate because they lack part of their genome. Therefore, their replication depends on co-infection with the wild type infectious virus, and their defective parental genome generates identical defective progeny genome copies. In cell culture, the presence of a high proportion of DI particles in an inoculum has been demonstrated to interfere with IPNV replication and progeny production, creating persistently infected monolayers [4]. Marjara et al. [68] also recently demonstrated—in persistently infected cells—the upregulation of genes involved in transcription repression, which suggests that IPNV persistence could be due to the reduction of viral replication as a result of a reduced transcription capacity. Other authors [59,69] have added that the persistence status could be due to the virus and host reaching a balance in such a manner that the virus reduces its level of replication so as not to harm the host and thus not triggering the host defenses.

## 3. The Disease

To analyze the mechanism of virulence modulation in IPNV-like viruses, it is also essential to understand the characteristics of the disease, the route of viral transmission and infection, and the host defense system. 

### 3.1. Viral Transmission

Due to the demonstrated stability of aquabirnaviruses in a wide range of temperatures, pH, and salinity, the virus can survive for long periods of time in a variety of environmental scenarios and different types of reservoirs (see review [70]). If we add its high capacity to infect a host—just 1 TCID_50_/ml of virus in a water tank is enough to infect a salmon—it is quite understandable that the virus is very efficiently transmitted horizontally, via water. Similarly, the high efficiency of the vertical transmission of the virus is also well known [4]. The survivors of an infection become asymptomatic carriers of the virus, even for years, acting as reservoirs of the virus and spreading it through the water—via feces—mainly during stress episodes, but essentially as breeders, through their reproductive products [5]. The ways the virus is able to persist in fish have been partially dealt with in Section 2.5, but only in relation to viral replication. However, another explanation for persistence comes from the ability of IPNV to enter blood leucocytes, escaping host immune defenses and keeping a low replicative profile to reduce damage to the host, as described below.

### 3.2. Characteristics of the Disease

In horizontal transmission, the virus enters through the gills, the intestinal epithelium and/or certain areas of the skin [4,62] via the surrounding water. Within few days the virus is detected in several tissues, and because it has been detected in circulating leucocytes, it is believed that the presence of the virus in the blood during the viremia phase is the reason behind the rapid distribution of PNV through most of the fish tissues, including kidney, spleen, pancreas, liver, heart, and brain as well as skin, intestine, and the reproductive cells. To this regard, although there is a consensus that maximum replication is reached in the head kidney, and during acute infection also in the intestine and pancreas, in some cases the virus has been detected in other organs than the pancreas. In fact, it seems that tissue tropism depends to a high extent on the fish species, the viral strains and even the age of the fish; the most susceptible age corresponds to very young fry, where the virus can replicate to high levels in most tissues.

The most characteristic symptoms include the unusual behavior already described above, typical of some neurotropic viruses, as well as anorexia; but also includes more general signs of skin darkening, exophthalmia and petechial hemorrhages in the ventral surfaces. Internally, the histopathological lesions include necrosis of the intestinal mucosa, progressive pancreatic acinar cell death, and damage of the hepatic tissue, although some of these lesions are not always visualized.

### 3.3. Cell and Host Defenses

Inside the host, the virus encounters immune defenses—at two levels—to unbalance the virus–fish interaction in favor of the host: the innate non-specific and the specific adaptive defenses. The innate defense system is quickly activated (in a few hours) to fight against any virus—actually, against any infection—but, because its prolonged activity can damage the host, this must use a second barrier, the adaptive immune response. This comprises the humoral response, which corresponds to the induction of antibodies against a specific antigen (a specific virus, for instance), and the cell-mediated immune response. Unlike the innate defenses, the adaptive response has a long-term effect; however, because it is specific, the response is slower than the non-specific one. There is an exception to this, because when the virus enters the bloodstream, a non-specific and non-induced circulating immunoglobulin (named 6S-serum factor due to its sedimentation coefficient) with certain anti-IPNV activity was demonstrated to be present in normal trout serum (not previously immunized rainbow trout, *Salmo gairdneri*) [5].

*Apoptosis*. As soon as the virus reaches the portal of entry, it has its first opportunity to replicate inside a susceptible cell in order to proliferate and spread, and the host has its first chance to avoid the massive proliferation of the invader. Inside the cell, IPNV will start the reduction of the cell’s DNA and protein synthesis in the first 4 h p.i. [62] to break the balance in favor of its own replication, but the simple presence of the dsRNA and certain viral proteins trigger a cellular process that will unleash a cascade of events ending in “programmed cell death”, i.e., apoptosis. With this mechanism, the infected cell self-destructs in benefit of the surrounding cells, to avoid the spread of viral progeny. In non-infected cells, the expression of the genes responsible for all the events associated with apoptosis is blocked by regulatory proteins such as those of the Bcl-2 family. When those genes stop being inactivated, the caspase cascade is activated, starting a series of events ending in cell death: segregation of the chromatin and condensation of the cytoplasm and fragmentation of nucleus and cytoplasm producing the characteristic “blebbing” into small cell fragments which are engulfed by macrophages. IPNV has been demonstrated to induce apoptosis through repression of the Mcl-1 gene, a member of the Bcl-2 regulatory family [62] and there is certain homology to this type of antiapoptotic cell proteins with some domains of the VP5 IPNV protein.

*The interferon*. Most virus-infected cells produce interferon (IFN) in response to viral infection. IFN is a cytokine, a group of regulatory proteins involved in intercellular signaling as part of an antiviral defense strategy. There are specific cytoplasmic (RIGI/MDA5) and ER (TLRs 3, 7, 8 …) host receptors that recognize ds- and ssRNA molecules (ssRNA molecules form secondary structures resembling the double-stranded formation), initiating transcription of IFN-genes. However, it is not produced during the transcription of non-infected cells, but only when large quantities of dsRNA are present in the cytoplasm, such as when the cell is infected with IPNV. IPN acts in different ways: first, apoptosis is activated in extended periods of IFN induction; in addition, IFN acts indirectly by inducing the expression of the major histocompatibility complex (MHC) genes and the natural killer cells to lyse the virus-infected cells, and directly, by the induction of the synthesis of specific antiviral proteins.

Among the most studied of these proteins in IPNV-infected cells is the Mx protein, which inhibits viral replication in certain fish cells in vitro, but has also been demonstrated to be produced in infected salmon [4,62]. Lockhart et al. [71] observed a high synthesis of Mx mRNA in post-smolt salmon (a highly susceptible age), but not in parrs, which could be due to the lower quantities of viral dsRNA present in fish tissues when viral replication is dramatically limited at non-susceptible ages. However, the in vivo correlation between the induction of innate antiviral genes and protection against the virus is not actually clear [52]. Another IFN-induced antiviral protein demonstrated in fish is the dsRNA-activated protein kinase (PKR) proenzyme, present in few inactivated copies in non-infected cells; when the IFN induced by surrounding infected cells attaches to the membrane receptor of those, it induces an increase in the production of new inactivated copies of PKR; thus, when the cell is infected, the presence of viral dsRNA immediately activates many copies of the proenzyme, which phosphorylate the eIf-2 translation factor, blocking protein synthesis and reducing the proliferation of the virus.

*The adaptive immune response*. It is well known that IPNV is immunogenic and that the VP2 capsid protein is mostly responsible for the induction of the synthesis of neutralizing antibodies [5]. The detection of anti-IPNV neutralizing antibodies after the infection of fish is possible a few weeks post-inoculation, and they can be detected for some years in the survivors. It has been demonstrated that sera from immunized fish can protect naïve specimens against infections [72]. However, it seems that the immune response is not strong enough to protect fish against the disease, and immunized fish can show symptoms after episodes of stress. In fact, as Julin et al. [73] reported, in spite of extended vaccination against IPNV in Norwegian salmon farms, there are new outbreaks every year; therefore, it seems that the specific antibodies are not able to completely remove the virus from the fish. To this regard, the capacity of the virus to hide (and replicate at a low level) inside the fish leucocytes [69] could be the reason for evading the fish immune response.

### 3.4. Persistence and Immunity

As already introduced in Section 2.5, persistence is an important issue in IPN, since life-long persistently infected asymptomatic carriers shed the virus and thus represent a risk, and are responsible for creating endemic areas [73]. This status of persistence depends on the balance between the level of viral replication and the effectiveness of host defenses [69], and the inhibition and/or activation of certain cytokines is involved. To this regard, Reyes-Cerpa et al. [74] demonstrated the up-regulation of certain anti-inflammatory cytokines and down-regulation of other pro-inflammatory ones during IPNV acute infection, which was also observed in persistently infected fish. Marjara et al. [68] found that, during persistence, virus transcription was suppressed due to the up-regulation of host genes involved in the synthesis of transcriptor repressors and in viral protein degradation, which reduces (if not eliminates) viral replication. In fact, it is obvious that the interferon cell defense system is used by the host to block viral replication and spreading; but, for the virus to persist, it must be able to reduce its own replication to a minimum level in order to limit the IFN response [69]. This can be done by increasing the proportion of DI particles, by the selection of variants with lower replication capacity among the infecting quasispecies present in the host, or by hiding from host defenses by entering the leucocytes. The capacity of the virus to survive and even replicate—to a certain extent—inside the fish leucocytes has been widely reported [4] and makes IPN an immunosuppressing-like disease [75], with bacterial infections frequently associated to the presence of IPNV [62].

## 4. The Concept of Virulence and Parameters Influencing It

**Figure 5:**
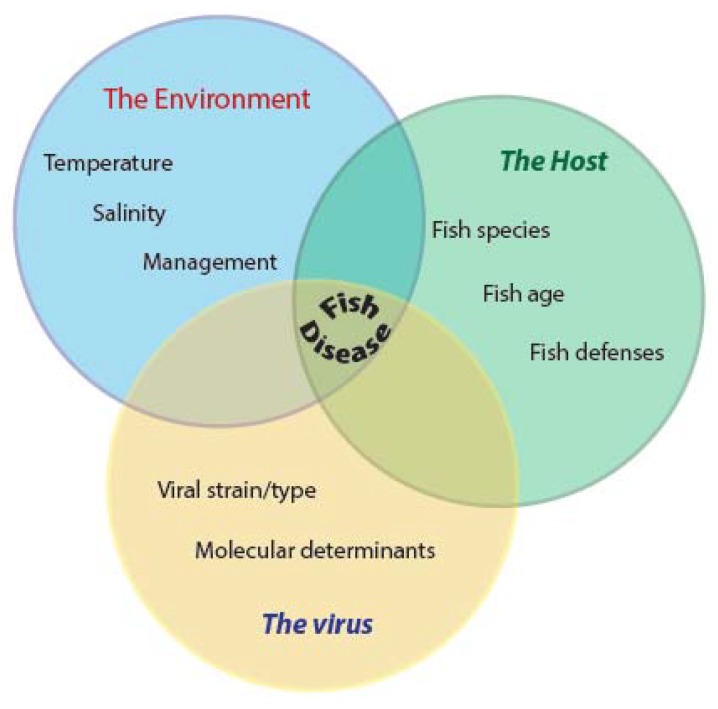
Factors modulating virulence of the IPN disease (Adapted from Snieszko [77]).

Now that we are coming to the main objective of this review—the parameters determining the virulence of the IPN virus—we must remember some basic concepts of pathogenicity. The first is the definition of pathogenicity itself, a term which means the capacity of an agent to cause disease in a host; and second, the term virulence, occasionally used incorrectly in some reports, is defined as the level of adverse effects a certain pathogen exerts on a host. No doubt IPNV is pathogenic in salmonid fish; it is not high or low pathogenic, but high or low virulent in a specific host, under a specific set of external conditions, and in a specific environment. This takes us to the well-known diagram designed by Snieszko in 1973 [76] to describe the parameters influencing the development of a disease (Figure 5), which we will use to address each issue involved in the development of the IPN disease, and, that makes it possible to predict the level of virulence of this virus in an episode.

### 4.1. The Environment

Due to its high stability under environmental conditions, IPNV is a ubiquitous virus. It has been isolated from cold- (≤20 °C), cool- (20–28 °C) and warm-water (>28 °C) scenarios worldwide [70,77] and from both asymptomatic and diseased fish. Although the optimum temperature was reported to be between 10 and 15 °C for the virus to develop disease in salmonids [8], it has also been isolated from outbreaks in waters between 5 and 25 °C [77]; the wider ranges are reported in Asia (5–15 °C in Middle Asia, and 12–25 °C in the Far East), although in Europe it has also been isolated from diseased fish at temperatures as high as 22 °C. This means that the virus has the capacity to adapt to almost any water temperature, but it is also known to be able to survive in a wide range of salinities, from 0‰ (freshwater farms and rivers) to 40‰ (from estuaries to sea water facilities and wild populations). 

The most crucial environmental conditions affecting the development of the disease are related to human management and, more precisely, to stress and transmission route management. Reducing the risk of introducing the pathology in IPNV-free areas requires the correct regulation and implementation of biosecurity strategies, related to horizonal transmission (zoning of areas/farms by their level or risk, controlling stock movements, surveillance and monitoring of wild and farmed stocks, eliminating IPNV-infected stocks on farms, and disinfecting affected facilities) and vertical transmission (non-lethal selection of IPNV-free adult wild fish before introducing them into farms as breeders, monitoring breeder stocks to remove any suspicious individual, and never use survivors of the disease as future breeders). On the other hand, stress is an important parameter influencing the disease, and in the farming system it depends on the physicochemical conditions of the water, inappropriate nutrition, manipulation of the fish, and the density of the fish population in the facility. To this regard, the excessive density of cultured fish, a very important and often ignored parameter, has a double effect on the artificial environment in farms: on the one hand, it provokes stress in the fish, which is known to affect host defenses and has been reported to trigger outbreaks in asymptomatic carriers [78,79]; on the other hand, high fish densities favor virus transmission and virus evolution to high virulent types [80]. Therefore, it is clear that fish density represents a very important parameter in fish mortalities by IPNV.

### 4.2. The Host

Obviously, the host is also a very important element in the development of the disease, which is influenced by fish species, fish age, physiological conditions, and even the genetic characteristics of the individuals. It is well known that the species most susceptible to IPN disease are salmonids, mainly rainbow trout and Atlantic salmon [4], in which the virus produces the most frequent and characteristic symptoms. However, as already described above, IPNV has been isolated from diseased fish from other species such as European and Japanese eels (*Anguilla anguilla* and *A. japonica*, respectively), in which it produces necrosis of the gill lamellar cells, yellowtail (*Seriola quinqueradiata*), where the virus is known as the yellowtail ascites virus, and flatfish such as turbot (*Scophthalmus maximus*) and Senegalese sole (*Solea senegalensis*) with external symptoms like those of IPN [4]. The age of the fish is also an important determinant of the virulence of the virus and of the level of mortality; although young fish of different ages can be affected, the most susceptible ones are very young fry, which can suffer very high mortalities, even close to 100%.

The capacity of the host to defend against virus invasion and proliferation, its immune response—described in Section 3.3—depends, to a large extent, on its physiological state, and stress has a great influence on it and hence on the reduction of the effectivity of the defense barriers and on the development of the disease [78,79,81]. Another approach to understand the variable susceptibility of fish is more related to genetics, since it has been demonstrated that different lines of the same species, with the same age, and under the same conditions, can respond differently to infection of the same viral strain. Therefore, there have been attempts to identify quantitative trait loci (QTL) associated to the level of susceptibility or resistance to the virus of rainbow trout and Atlantic salmon [82,83].

### 4.3. The Virus

The third component of the trio of factors defining the disease corresponds to the virus itself, and the virus-related determinants which define the level of virulence and are involved in the development of the disease with lower or higher adverse effects on the host. Since this is the main aim of this review, the following sections will look into the different approaches that scientists have made to point out a single virulence marker or to discover all the viral determinants involved in the level of virulence, as well as the first attempts to define markers to predict the level of risk of new isolates, the deep analysis of genome and proteins, and also the mechanisms of replication, inheritability, variability, and adaptation.

## 5. Virulence Markers of IPNV

### 5.1. First Attempts to Find Virulence Determinants

*Viral types and virulence*. As expected from the wide variety of IPNV types (Section 2.1), aquabirnaviruses are very diverse; in fact, aquabirnaviruses are much more diverse than avibirnaviruses, for which only two IBDV serotypes have been described. Blake et al. [13] applied phylogenetic analysis to a number of IPNV strains and observed a certain relationship between types and geographic origin. This was thought to help organize the IPNV epidemiological map, since by then a certain relatedness between the geographic origin of the strains and level of virulence was accepted: the American WB strain (genotype 1) was supposed to be of high virulence and, among the most represented European strains, Ab (genotype 2) was considered of low virulence and Sp (genotype 5) of high, and responsible for most outbreaks in Europe [81,84,85]. However, on the one hand, isolates from genotype 2 produce disease in rainbow trout on inland farms in Finland [86] and, on the another, A1 strains can produce low to high mortalities in brook trout (*Salvelinus fontinalis*) in North America [8]. To make things even more complicated, just like IBDV and many other RNA viruses, IPNV exists as a quasispecies; therefore, it is understandable that in each single strain a large range of variants may coexist, making the virus very adaptable to new environments and conditions [87]. The fact that there are different levels of virulence of different strains within the same type has been well known for decades, and this has made scientists focus on other parameters to understand how virulence is modulated.

*Viral replication*. Directly or indirectly, the capacity of a viral strain to replicate in cell culture has been considered as a marker of its in vivo virulence. In 1973, Vestergård-Jorgensen [88] discovered that non-infected rainbow trout serum (RTS) inhibited IPNV replication in cell culture, and soon other researchers observed that after several passages in a cell line, the cell culture adapted (CCA) strain were more susceptible to RTS than the original one [89]. Since the CCA strain provoked lower mortalities in challenged fish, it was initially postulated that the capacity of a strain to replicate in the presence of RTS was synonymous with high virulence in vivo, but there were reports supporting the assumption and others against it. Finally, Park and Reno [89] demonstrated that there was no relationship between both parameters.

Another approach was the assumption by Dorson et al. [90] that the level of virulence of IPNV was related to the size of the lysis plaques in infected monolayers, and thus on the in vitro replication capacity of the virus. However, it was soon found that although certain relationship between replication and virulence might exist, there really is no correlation between virulence and plaque size [91,92].

Directly or indirectly, viral replication also has something to do with virulence. The statement by Nagarajan and Kibenge [93] that “the replication ability of the [IBDV] virus has an influence on its virulence” must be true also for IPNV. But, for IPNV this statement must be qualified since it has been demonstrated when viral replication is measured in vivo, not in vitro [52]. In fact, the observations in vitro are completely different: CC adapted strains, which produce high viral titers in cell monolayers, behave as persistent or even non-virulent in vivo. Nevertheless, even in vivo some discrepancies exist [81].

*Viral genome or proteins?* In the same report by Sano et al. [92], where they deduced that plaque size did not determine the virulence of IPNV in fish, they also showed that plaque size was actually associated to segment A. In another study by the same group [94], the authors concluded that segment A was also involved in cell tropism and virulence. The participation, almost exclusively, of segment A in the virulence of IPNV has been suggested by several authors [21,80]; however, as we will see below, the involvement of segment B cannot be denied. A similar discussion existed with IBDV, until Nouën et al. [61] clearly confirmed the participation of both segments in the modulation of virulence. The genomic segments not only participate in virulence generating the viral proteins where the determinants of virulence are located, as will be shown in the following sections. But also, their compatibility with each other is important for an efficient replication [61] and, in addition, as already described above, the 3′ and 5′-UTRs of both segments have important signals for several steps of viral replication [93]; in both cases it is clear that the final effect would be reflected in the level of virulence to the fish.

### 5.2. Determinants of Virulence in VP2

**Figure 6:**
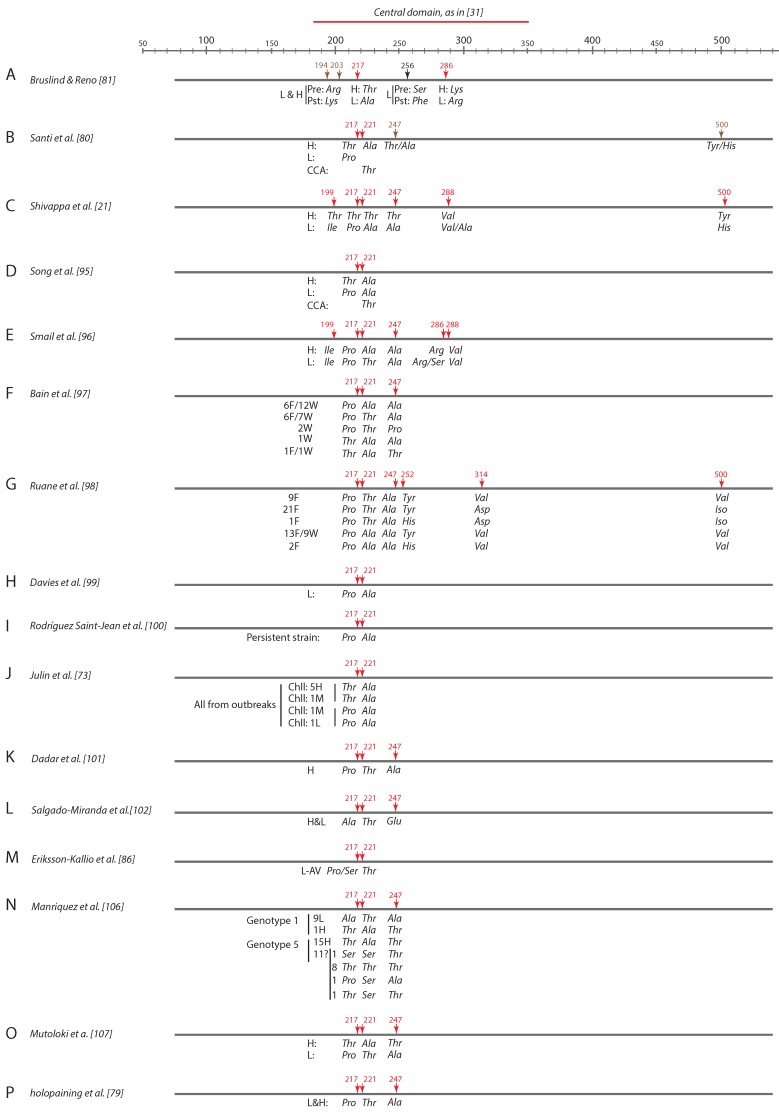
IPNV virulence determinants in VP2.

VP2 has been considered the most important viral protein implicated in virulence. In 2000, Bruslind and Reno [81], studying the association between the virulence in brook trout fry of three Buhl strains of IPNV and their VP2 and VP3 aminoacidic regions, observed no differences in VP3, and some very specific ones in VP2. There were three groups of differences in this protein (Figure 6A): (i) at the amino acid (aa) positions 217 and 286, the strains of low (L) virulence in vivo had an alanine (Ala) and the high (H) virulent a threonine (Thr); (ii) the second difference was between phases pre- and post-peak of mortality, since both L and H strains changed from arginine (Arg) to lysine (Lys) in the aa positions 194 and 203; (iii) in position aa_256_, some changes were observed before and after the peak, but only in one of the L type strains (Figure 6A). The authors pointed at aa_217_ as most probably being involved in virulence, since the change from a polar (Thr) to a hydrophilic (Ala) aa might hinder adsorption, whereas the changes in the remaining positions did not seem to be relevant.

Santi et al. [80], working with a different strain (Sp), found most changes between attenuated (cell adapted), low, and high virulence in VP2, and did not consider VP1, VP3, or VP4 in spite of the fact that some minor differences had also been observed in these genes (0.2, 1.7, and 0.9%, respectively). They also found differences in VP5, which will be discussed below. In VP2, they found four motifs, located at aa 217, 221, 247, and 250 (Figure 6B), although they considered mainly aa_217_ and aa_221_. Motif 221 coincided with that described by Bruslind and Reno [81], and in both cases the aa Thr was identified as a virulence determinant; however, there were differences between both reports with respect to the markers of low virulence because those authors found Ala to be the determinant, whereas for Santi et al. [80] it was Pro. For the latter, a second important determinant was located in position 221, and a substitution of Ala by Thr denoted a change from virulent to cell culture-adapted or attenuated virus.

In the same years [21], three SP strains of high (79%), medium (46%), and low (16%) mortalities in post-smolt Atlantic salmon were sequenced, and mutations in 13 positions were found, six in VP2, two in VP3, and five in VP1, but they only considered those in VP2 as putatively related to their level of virulence (Figure 6C). They found two new positions (aa_199_ and aa_286_) which seemed to be determinants to differentiate L from H strains. Surprisingly—because some of the authors were also authors of the previous report—there were certain discrepancies. The only one which coincided was aa_217_ (Thr in H strains and Pro in the L ones); for the rest, the virulence determinant did not coincide: Thr–Thr–Tyr (aa_221_–aa_247_–aa_500_) for the H strain in the second report, and Ala–Thr/Ala–Tyr/His in the previous one. Interestingly, in aa 247 and 500 the second alternative for H type in the previous report was characteristic of L strains for Shivappa et al. [21].

In another report by Song et al. [95], the same group made an effort to simplify and standardize the markers of virulence in Sp strains (Figure 6D). They generated recombinant strains using the backbone sequence of a virulent strain and substituting residues at positions 217 and 247 for the amino acids present in L strains. Their recombinants developed low mortalities in challenged fish, which demonstrated that both residues could be used as markers of virulence, at least in the case of Sp strains. Additionally, they applied 10 passages in cell culture of a virulent strain and observed that the significant reduction of mortality was associated with a single substitution of Ala for Thr at aa_221_. They concluded that amino acids 217 and 221 were determinants of virulence for IPNV Sp strains and that the markers of high virulence were Thr/Ala (aa_217_/aa_221_) and Pro/Ala of moderate to low; in addition, a Thr in aa_221_ was proposed as a marker of avirulent (attenuated) strains.

Smail et al. [96] reported a study also working with Sp strains, in this case isolated from the Shetlands (from outbreaks: high virulence) and Scotland (from asymptomatic carrier fish: low virulence). The results, summarized in Figure 6E, showed that the positions with changes between L and H strains coincided with those of previous reports [21,95] but the virulence determinants were completely different. In fact, using the determinants proposed by Song et al. [95], the H Shetland strains should be of low virulence. They concluded that the interaction between the viral strains, the fish species and even the geographic origin could be quite complex and therefore “it may be premature to define certain residues as vital determinants”.

Bain et al. [97] sequenced a large number (36) of IPNV isolates from farmed and wild Atlantic salmon in Scotland, and reported 5 profiles of residues 217, 221, and 247 (Figure 6F). They found a profile of expected high virulence (Thr–Ala–Thr) according to Song et al. [95] and Shivappa et al. [21] not only in farm derived fish but also in wild fish. Remarkably, they found IPNV strains isolated from outbreaks with the Pro_217_–Ala_221_ profile (characteristic of low virulence) instead. They concluded that not only the interaction of other factors but also different genes might be involved in modulating virulence.

In 2009, sequencing 55 Irish isolates mostly from outbreaks in farmed Atlantic salmon, Ruane et al. [98] found five different series of motifs corresponding to the VP2 aa positions 217, 221, 247, 252, 314, and 500 (Figure 6G). They observed that all the profiles included the aa Pro at position 217 and a large number had Thr at aa_221_, determinants of moderate to low and avirulent strains, respectively, according to Song et al. [91], although most corresponded to outbreaks and they were therefore expected to be H. However, using the prediction by Smail et al. [96] instead, only 15 of the strains from the farmed fish would give a correct result. Nevertheless, other authors [99,100] found a correct correlation between the observed virulence of their IPNV strains and the profiles reported by Song et al. [95] for the low (Pro_217_-Ala_221_) and attenuated or avirulent (Thr_221_) strains.

In an interesting study by Skjesol et al. [52], the authors used two Norwegian IPNV strains isolated from diseased salmon with the VP2 markers profile of H strains according to Santi et al. [80] and Song et al. [95]; however, those strains had been isolated from cases of moderate (32%) and low (5%) mortalities. Before challenging Atlantic salmon, they were subjected to 2–3 passages in cell culture, and before the infection they were sequenced again to check their profile, observing that they had mutated to Thr_217_–Thr_221_–Thr_247_ and Thr_217_–Ala/Thr_221_–Thr_247_. This means that one of the strains had moved to an avirulent (AV) type of strain (due to the presence of a Thr in aa 221), and the other had passed to be a mixture of H (Thr–Ala–Thr) and AV (Thr–Thr–Thr) variants. Interestingly, after the challenge, the recovered strains were both L type (Pro–Ala–Ala).

In 2013, Julin et al. [73] isolated and sequenced the VP2 gene of 18 Sp IPNV isolates from Norwegian Atlantic salmon farms during outbreaks. They observed that, although expected to be of high virulence, some of the strains showed the L profile (Pro_217_-Ala_221_) according to Song et al. [95]; therefore, they performed a challenge with some of the strains and observed that those with the Pro–Ala profile became of low or moderate virulence in the challenge, which confirmed the prediction (Figure 6J). In addition, the authors subjected a reference H strain to passages in cell culture and confirmed not only its attenuation in vivo, but also the substitution of aa_221_ from Ala to Thr, as expected [95].

Moving on to Asia, Iranian Sp IPNV isolates obtained from cultured rainbow trout were demonstrated to have the Pro_217_–Thr_221_–Ala_247_ profile [101] (Figure 6K), which disagrees with Song et al. [95]. In Mexico [102], IPNV isolates showed Thr at position 221 (Figure 6L) which is indicative of persistence; however, although most isolates were from asymptomatic carriers—which confirms the predictions—one corresponded to an isolation from diseased rainbow trout. Something similar was reported in Chile [103], Turkey [104], and Taiwan [105], with isolates from clinical cases showing Thr at aa_221_. More recently, a discordance was also observed by Holopainen et al. [80] in Finnish strains (Figure 6P); they sequenced isolates obtained during a few decades and observed that all of them showed the aa Thr at position 221, in spite of the increasing virulence observed over recent years in Finish farms. Julin et al. [69] tested the capacity of two strains of H and L virulence—with the corresponding determinants of virulence in aa_221_ (Ala for H, and Thr for L)—and observed that during the freshwater phase both viruses could attenuate the host response persistently infecting and not harming it. However, other reports do not show the failure of the markers proposed by Song et al. [95]. In Chile, Manriquez et al. [106] reported a correspondence between the observed and the expected profiles (Figure 6N) as in Finland by Erikson-Kallia [86] and in Norway by Mutoloki et al. [107]. Nevertheless, it is obvious that there are too many discrepancies to accept that just a single profile of aminoacidic residues of VP2 can be used as a reliable marker of the level of virulence of the virus and, as suggested by other authors, other genes must also be involved in modulating virulence. We will now address one of the most studied: the VP5 protein.

### 5.3. Is VP5 Expression a Determinant of Virulence?

As already described in Section 2.3, although the involvement of VP5 in IBDV virulence seems very clear [56], favoring the circumvention of the early host defense mechanism and viral spreading thanks to the inhibition of early apoptosis, in IPNV the function of this protein is a source of controversy.

Several authors ensure that IPNV–VP5, as in IBDV, is an antiapoptotic viral protein, which blocks early apoptosis allowing the virus to spread through host tissues, develop disease and generate high mortalities. Hong et al. [58] first demonstrated that IPNV induces apoptosis in infected cell lines, and later, using cloned VP5, reported that this viral protein shares the BH1, BH2, BH3, and BH4 domains of the Bcl-2 protein family as well as its antiapoptotic function. They also suggested that the overexpression of VP5 induced a host apoptotic-off system to enhance progeny production [55]. The results by Skjesol et al. [60], showing that VP5–IPNV inhibits IFN signaling, increasing virus replication, seem to support this assumption. In addition, Santi et al. [80], studying several IPNV isolates, observed a two week delayed mortality curve associated with the unique strains lacking VP5 ORF (in comparison with the remaining strains they studied, which had the 12 KDa truncated version); to our understanding, this could mean that the absence of the VP5 protein reduced the capacity of the virus to spread within the host and between individuals, which would support the assumed function of VP5, as a way of modulating virulence.

However, the same group soon reported a different statement. Santi et al. [54,108] constructed three IPNV recombinants: rNV115, based on the Sp type field isolate NV-115, corresponding to the wild type recombinant and which express a 12KDa truncated VP5; rNV115-15K, a recombinant expressing a full length 15 KDa VP5, and rNV115-Δ VP5, a recombinant which does not express the protein. They observed: (i) no differences among the growth curves of the three recombinants in CHSE-214 monolayers; (ii) no differences in cumulative mortalities in challenged Atlantic salmon post-smolt, and (iii) no differences in viral replication in the infected tissues. Regarding apoptosis, in a first assay the authors observed a slightly higher percentage of apoptotic cells in the monolayers infected with the non VP5-expressing ones, in comparison to the others, which suggested a certain influence of VP5 expression in the inhibition of apoptosis. The authors discovered that a certain proportion of the mutants used in the experiment had an aminoacidic substitution at position VP2 aa_221_, from Ala to Thr, which supposedly adapted the virus to replication in cell culture. Since it could have influenced the results in the assay, they repeated it using Thr_221_-free mutant recombinants, and observed no differences in the percentages of apoptotic cells between the VP5-expressing and non-expressing recombinants. Therefore, they concluded that the induction of apoptosis in infected cell cultures, as well as in fish, does not depend on VP5 expression, and finally stated that VP5 has no influence on IPNV in vitro and in vivo replication and virulence (and persistence). However, they also recognized that certain “substitutions of putatively important amino acids in the BH2 domain of VP5 […] might influence its anti-apoptotic effect […] and partially affect […]” the result. Nevertheless, other authors have reached the same conclusions regarding the lack of influence of IPNV–VP5 on both viral replication [18] and infectivity [57].

Another point of discrepancy is the effect of the size of this protein on its action. Although for some authors VP5 activity does not depend on its size, and even though apoptosis is not dependent of that protein [54,57,108], others defended the anti-apoptotic activity of the protein [52] and stated that a deletion in the 15 kDa normal VP5 protein might affect its activity and, therefore, viruses expressing a truncated 12 kDa VP5 should be more benign. To this regard, Davies et al. [99] noted that all the Australian strains from healthy fish that they sequenced had a deletion at the N-terminal; however, other authors found that differences in VP5 size in field isolates were not related to the level of disease [79,109]. In IBDV, a clear association between the size of the protein and the virulence of the strain has been demonstrated in field isolates [61]; thus, the very virulent IBDV strains exhibit a large ORF and the low virulent ones have shorter forms. However, the authors reported that those differences were not observed in recombinants with each VP5 type. In the case of IPNV, Julin et al. [73], using field isolates expressing VP5 proteins of different lengths (15, 12, and 3.3 kDa) in challenges, observed that strains of different VP5 lengths generated no differences in mortality, and others with VP5 of equal length provoked different mortalities. Unfortunately, they did not clarify the criteria whereby, for example, 51% is accepted as high mortality whereas 41% is not; in addition, since many of the isolates were demonstrated to be actually a mixture of variants (regarding VP5 presence/absence and length), the interpretation by the authors of this result ensuring that VP5 is not essential in virulence is, to my understanding, a little risky.

There are two possible explanations for all of these discrepancies. On the one hand, Ulrich et al. [59] suggested that VP5 could be a de novo gene, generated by overprinting on segment A, and providing an anti-apoptotic capacity and hence an advantage for the virus to be able to adapt to farmed vaccinated hosts and maintain a low level of replication in that environment. On the other hand, it has been demonstrated that not only VP5, but also the remaining viral proteins, except VP1, inactivate IFN signaling by antagonizing IFNa1 promoter activation [48] and it is well known [110] that the IFN system is involved in the activation of apoptosis. Therefore, the apparent lack of influence of VP5—absence or presence—to block apoptosis could be due to the fact that such actions might be perfectly covered by other viral proteins. It could even be that the apparent limited capacity of IPNV induction of apoptosis [57,111] was due to the fact that not one but several of its viral proteins are involved in limiting apoptosis.

### 5.4. Other Viral Proteins and Their Interactions are also Determinants of Virulence

Just considering that all viral proteins are involved in the activation (VP1) or blocking (the remaining) of IFN signaling, as demonstrated by Lauksund et al. [48], it is understandable that all viral proteins must be determinants of virulence. However, the focus to find the molecular determinants of virulence has actually been put on the former two proteins, and the remaining have been disclaimed, arguing that there were more substitutions in VP2 and VP5. Looking for the differences in the genome sequence of three Sp IPNV strains of high (H), moderate (M), and low (L) virulence in challenged Atlantic salmon post-smolt, Shivappa et al. [21] discovered aa substitutions in just 13 positions, 6 in VP2, 2 in VP3, and 5 in VP1 genes. In spite of showing that positions 968 (VP3), 187 (VP1), and 690 (VP1) of the L strains had a different aa residue than those shared by the H and M strains, the authors considered “molecular determinants of IPNV virulence [residing only] in segment A, especially in the VP2 region”; however, they recognized that other factors (as VP1) might also play a role. Similarly, Santi et al. [80], working with nine strains of type L or H, did not consider the minor differences encountered in VP1, VP3, and VP4 as candidates to be determinants, and only pointed to specific positions in VP2 and the size of VP5. More recently, Mohr et al. [109] reported that, in the appearance of virulent IPNV strains from previous isolates causing no disease in Tasmanian rainbow trout farms, only a change in VP3 was common between the isolates; however, since until then “substitutions in VP3… had not been attributed to change in aquabirnavirus virulence”, the authors considered it improbable that such a single change could be responsible for modulating virulence.

As described in Section 2.3, VP3 is an important protein in the replication cycle due to its interaction with the viral RdRp (VP1) and the genome [39,41]. Therefore, depending on its location, a single aa substitution altering an interaction domain might indeed affect the replication capacity of the virus and its virulence. For instance, in IBDV it has been demonstrated that mutations in the C-terminal end of VP3 might affect its interaction with other proteins [41]. In addition, VP3, through the constitution of the VP1–VP3-genome RNP, protects viral RNA from cell defenses [39], counteracting the IFNa1 promoter activation by VP1; failure in VP3 interaction with any of the other components in the RNP could compromise not only replication, but also viral RNA stability. VP3, by interaction with pVP2, also promotes the packaging of the viral RNA and the assembly of pVP2 to construct the capsid [46]. Therefore, it is risky to belittle the importance of aa substitutions in this protein.

In IBDV, Nouën et al. [61] discovered specific aa differences between H and L strains, which were located near the catalytic domain (in the central area) of the RdRp (VP1) viral protein, and they concluded that certain regions of the polymerase might be essential in virulence. The N-terminal of VP1 has also been reported as an important determinant of genome attachment [63] and a change in that end might affect interaction. Interestingly, the changes found by Shivappa et al. [21] in VP1 were located towards the N-terminal and in the central area.

Finally, the VP4 function is not only linked to its protease activity (the failure of which would affect the processing of the polyprotein and hence viral production), but it is also a potent IFN induction inhibitor (not associated to its protease activity), and in IBDV it has been demonstrated to trans-activate the synthesis of VP1 [45]. Again, it is understandable to consider VP4 as a bearer of some virulence markers.

## 6. Other Genetic Markers

*Reassortment*: During coinfection of a cell with two variants of a viral strain, several recombination phenomena can occur which can provide diversity, adaptation and even the possibility to survive from lethal mutations. Among these recombination mechanisms, reassortment is the most common in segmented viruses and is known to be one of the most involved in the evolution and diversity of RNA viruses [112,113]. In a segmented genome, each segment codifies a gene or a few genes and, therefore, is responsible for the specific phenotypic characteristics of the virus; the possibility of a viral progeny sharing phenotypic characteristics from two or more parental variants gives rise to new infection capacities, and tropism, and new antigenic forms [114,115], which has an obvious effect on pathogenicity. This phenomenon has been demonstrated to occur in several segmented fish viruses, and aquabirnavirus, of segmented genome, was also expected to reassort. Regarding IPNV, although reassortment was reproduced in vitro in the 1990s [92], the first report on natural reassortment was in 2001 by our group [116], and many subsequent publications have demonstrated that the phenomenon is not scarce in nature. In IBDV, reassortment has played a demonstrated role in the appearance of very virulent strains and even of chimera viruses due to the coinfection of wild strains with attenuated vaccine virus [117,118]. In IPNV, apart from the study by Sano et al. [92] in the 1990s, there have been no attempts to clarify if reassortment was also implicated in virulence, and only some clues have been reported in a symposium [119]. Therefore, our group designed a study to discover a relationship between reassortment and cell adaptation and virulence [120]. The results confirmed that reassortment did provide the virus with a higher capacity to adapt to cell types, but although some association with different virulence was observed, the results to this regard were not conclusive. Therefore, it was concluded that other mechanisms must be involved as well.

*Polyploidy*. As already indicated in Section 2.2, IPNV has been demonstrated to be polyploid, and to package 1, 1.5, and 2 genomic equivalents [11]. The phenomenon was first reported to occur in IBDV by Luque et al. [121] who also demonstrated that the phenomenon could have a certain influence on the level of virulence of the virus in vivo. Our group not only demonstrated that the phenomenon also occurred in IPNV, but also that it had a clear influence on the replication capacity of the virus, because high titers were obtained from the fraction with more genome equivalents. Unfortunately, as with reassortment, the in vivo results were not conclusive: although the fraction with viral particles with more genome equivalents did show the highest level of virulence, the level of virulence of the intermediate fractions did not directly correlate with their number of genome equivalents.

## 7. Conclusions

If with this review we expected to find out which marker, or set of well-defined markers, could be used to predict the level of risk of a new IPNV isolate from its molecular characteristics, by now we must be a little disappointed. In addition to the strong influence of the host (species susceptibility and host defense) and environment (adverse environmental conditions affects to both virus and host), the important influence of the virus itself is also well known, but there is no a single viral determinant of virulence. The viral genome and proteins, the interaction between them, a bunch of specific amino acid residues in VP2 (and indeed in the rest of viral proteins), the efficiency or failure of viral proteins to target specific host proteins (affecting the efficacy of the virus to control certain cell processes), reassortment, and polyploidy: these are all parameters combined in a way that, with the information available to date, is too complex for us to understand and hence to be able to discover such a clear, definitive, and simple track to predict virulence levels with some reliability. Many attempts have been carried out to discover such a magic marker but, in most cases the final conclusion of the researchers has been that other factors must be involved. The closest we have been was with the aminoacidic residues at VP2 positions 217 and 221. We thought it could be the magic marker of IPNV virulence; however, too many exceptions were uncovered. Then, it was thought that it could be applied at least in the case of Sp-type strains; but there were some exceptions again. Therefore, we must consider how a group of genetic factors are combined to determine virulence. That is why research should be aimed at the analysis of all mutations—in both segments, and including the non-coding regions—associated with different (and well defined) levels of virulence in vitro (infectivity, replication, and spreading) and in vivo (percentage of mortality in different fish species and under different environmental conditions).

## Figures and Tables

**Table 1:** Serotyping and genotyping of aquatic birnavirus.

Stp ^1^		Gtp ^2^	Type Strain	Geogr Origin ^3^
A	A1	1	WB	USA
	A2	5	Sp	Denmark
	A3	2	Ab	Denmark
	A4	6	He	Germany
	A5	3	Te	UK
	A6	3	C1	Canada
	A7	4	C2	Canada
	A8	4	C3	Canada
	A9	1	Ja	Canada
B	B1	–	TV-1	UK
		7	MaBV ^4^	Japan

1—Serogroup/Serotype; 2—Genotype; 3—Geographic origin; 4—Marine birnavirus.
